# CIRCULATING SASP FACTORS ARE ELEVATED IN PATIENTS WITH POSTOPERATIVE DELIRIUM

**DOI:** 10.1093/geroni/igad104.0327

**Published:** 2023-12-21

**Authors:** Allyson K Palmer, Thomas White, Elizabeth Atkinson, Nathan LeBrasseur

**Affiliations:** Mayo Clinic, Rochester, Minnesota, United States; Mayo Clinic, Rochester, Minnesota, United States; Mayo Clinic, Rochester, Minnesota, United States; Mayo Clinic, Rochester, Minnesota, United States

## Abstract

Aging, a major risk factor for delirium, is associated with accumulation of senescent cells in many tissues of the body. Senescent cells produce a host of proinflammatory and tissue remodeling factors collectively termed the senescence-associated secretory phenotype (SASP), which are secreted into circulation. The relationship between increased senescent cell burden and risk of delirium is not known. This study aimed to determine whether changes in circulating SASP proteins are associated with increased risk of postoperative delirium in cognitively intact older adults. Participants were recruited among patients scheduled for surgical or transcatheter aortic valve replacement, and blood was obtained at the presurgical visit. SASP factors were measured using commercially available multiplex magnetic bead immunoassays based on Luminex xMAP multianalyte profiling platform and analyzed on a MAGPIX system. Activin A concentration was determined by a Quantikine ELISA kit. Delirium cases were identified by manual chart abstraction, utilizing the problem list, ICD-10 codes, clinical notes, and nursing flowsheets. Eight of 62 (12.9%) of patients within the cohort developed postoperative delirium. Length of stay was significantly longer for patients who developed delirium (10.6 days) versus patients without delirium (6 days). Concentrations of SASP factors Activin A and PAPP-A were significantly higher in plasma of patients with delirium vs. those who did not develop delirium. IL-1 alpha concentration was significantly lower in patients with delirium vs without delirium. Other SASP factors measured were not statistically different between groups. More study is needed to determine whether these changes are directly related to increased senescent cell burden.

